# Editorial: Rowing: advances in training and performance—an editorial

**DOI:** 10.3389/fspor.2023.1248798

**Published:** 2023-07-14

**Authors:** Stephen J. Ives, Justin A. DeBlauw, Rohan Edmonds

**Affiliations:** ^1^ Health & Human Physiological Sciences, Skidmore College, Saratoga Springs, NY, United States; ^2^ Exercise and Sports Science, Macquarie University, Sydney, NSW, Australia

**Keywords:** rowing, athletes, fitness, assessment, equipment, technology, environment, training

**Editorial on the Research Topic** Rowing: advances in training and performance

While the history of rowing may date back millennia, the modern version of the sport was borne out of England in the inaugural Oxford-Cambridge boat race in 1829 (1). Since this formalization of competitive rowing there have been obvious evolutions in the sport, such as the formation of organizing and governing bodies overseeing the sport, namely the Fédération Internationale des Sociétés d'Aviron (FISA) and the addition of rowing to the Olympics. Since then, FISA, now “World Rowing”, has been a central governing body recognized by the International Olympic Committee (IOC). This background is critical considering that World Rowing sets official race distances, weight classes, and equipment standards, in efforts to ensure fair racing (1). Understanding this context is critical for coaches to accurately assess and train their athletes for competition, especially as the IOC and World Rowing have decided to accept a shortened course for the 2028 Olympic games (from 2,000 to 1,500 m).

Despite the history of rowing, and its firm establishment as a *bona fide* sport in the Olympic games, the study of rowing has been somewhat limited. From PubMed search of “rowing” the first entry is in 1868 investigating the “Effects of Rowing on the Circulation…”, which predates the first official soccer match (c. 1872). However, the number of articles netted from searching “Rowing[title]” in PubMed is 677, while the same search approach “Soccer[title]” yields an impressive 6,887 articles. Thus, with over a century of rowing training and competition, research on rowing pales in comparison to other sports.

**Figure 1:**
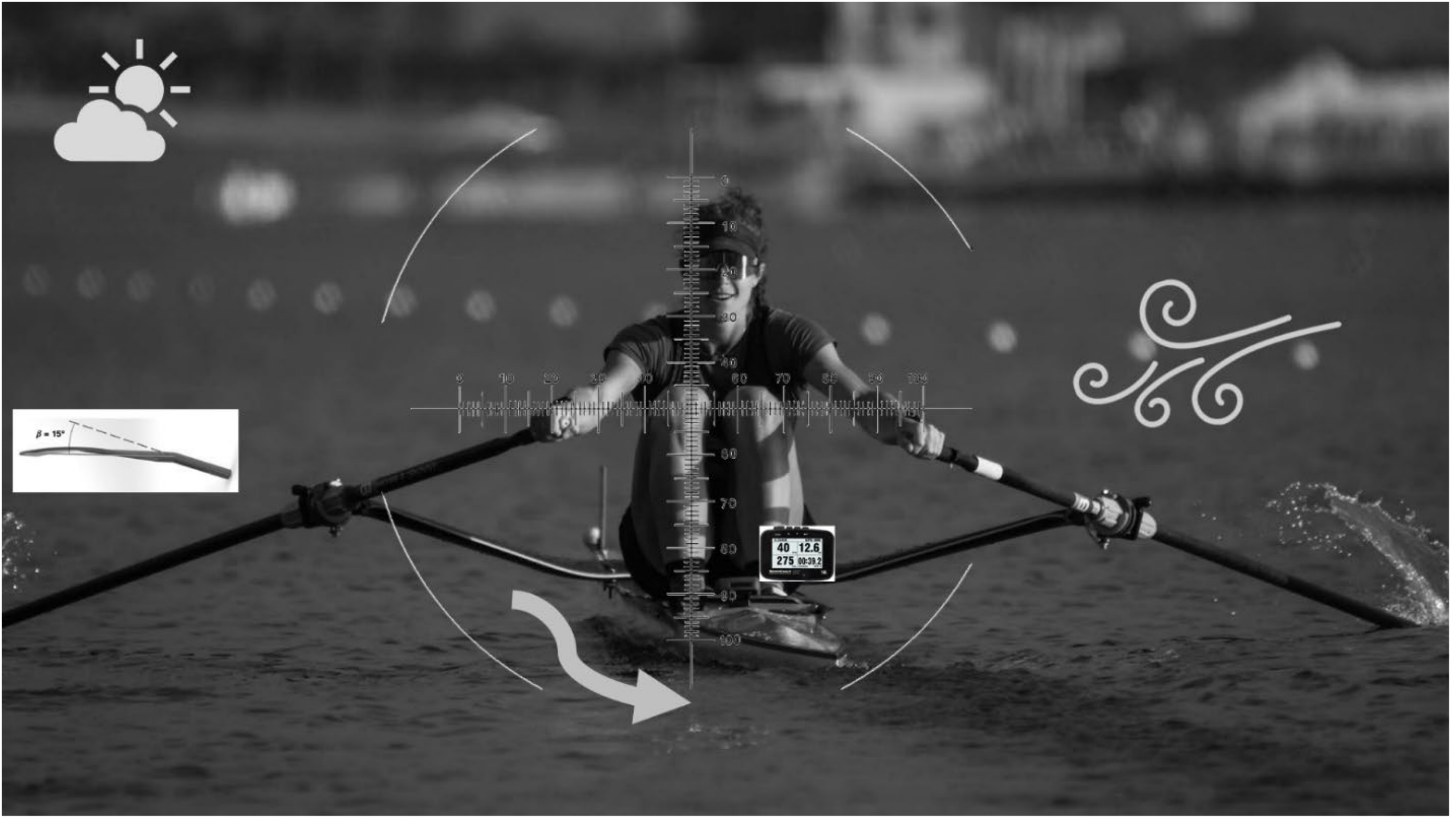
Schematic of the key factors of rowing performance, the athlete (e.g., anthropometrics such as height, sitting height, wingspan, etc.), the equipment (blade angle/size), race course/conditions (temperature, sun, wind, current, etc.), analytics (e.g., GPS Speed coach, NK), and the training (e.g., anerobic power reserve method) that induces adaptations to elicit high performance.

Accordingly, the present research topic sought to publish works on rowing: advances in training and performance. Rowing is a high-intensity endurance sport, demanding a large volume of training for anaerobic metabolism, aerobic capacity, as well as muscular strength, endurance and power (2). Though physiological attributes may be a good indicator of rowing performance (2), a better understanding is required on the importance of economy/efficiency (3), training methods, technique, nutrition, technology/equipment, and athlete monitoring/management strategies (4) and their relation to training capacity and/or performance. The articles within the present research topic published in the journal are an eclectic, but essential collection of articles addressing the key factors in rowing training and performance: the athlete (Busta et al.) equipment (Nieuwburg et al.) venue or course (and conditions) (Binnie et al.) and training aspects of rowing performance (Wang and Zhao) (Figure 1).

A critical aspect of research is the concept of translation, transferability, ecological validity, or application to real world scenarios. Given the logistical challenges with assessing rowers in outdoor, largely inaccessible situations, namely being out on rivers or lakes with both the rower and boat moving dynamically, have made situation specific assessments quite difficult, even for coaches. Since the advent of the rowing ergometer coaches and athletes have relied on this as a key metric, along with others such as “seat racing” or individual or crew time trials. Rowing machine performance may relate to on water performance, but coaches and athletes alike have long recognized this is an imperfect relation considering the intense technical nature of the sport.

Thus, one of the articles published in the topic (Binnie et al.) a perspective written by Binnie and colleagues nicely summarizes the current state of affairs regarding our ability to assess on-water performance in rowing. While the aforementioned off-water performance assessment on the rowing ergometer and its associated limitations are to be recognized as a standardized approach to compare performance of rowers all over the world; on the water comparisons are muddied by a myriad of factors. As indicated by the authors (Binnie et al.) while each rower or coach has their penchant for the variable they deem most detrimental to performance, likely the most foreign to home training environment; environmental factors such as heat/humidity, wind speed/direction, water temperature/depth/current speed/direction, course marking and straightness, and lane selection all likely interact and affect performance. However, other environmental factors such as altitude, uv index, air quality, distance from training home, and time allowed for training and acclimation at competition sites are also likely contributors to variability in on-water performance across the globe. As suggested by Binnie et al. the rise in technology and instrumentation may help to allow for better performance comparisons, but perhaps rowing can take a nod from F1 and instrument each boat/athlete providing real-time, and *post hoc*, analysis of relevant data (wind speed, water and land speed of boat, etc.) (Figure 1).

In a similar vein, Nieuwburg et al. used an innovative approach to understand how altering oar blade design might alter on-water performance. Using a scaled robotic model, the authors explored the impact of a 15° forward blade angle and altered blade size would alter mechanical efficiency, and indeed it was found that, all else equal, a modified rowing blade can improve boat speed by 0.4%. While this may sound paltry, at the 2022 world championships men's 1× final, such a difference could've brought the 4th place finisher into the bronze position. Testing in athletes is an obvious next step, though whether FISA would allow it may be another matter.

In terms of understanding the human factors contributing to performance Busta et al. explored anthropometric factors, such as typical height and weight, but also unique parameters such as handgrip strength, sitting height and arm span, and both normalized to body height, in heavyweight and light weight male and female rowers at the 2022 World Championships. The authors used a somatotype dimensional analysis to compare the athletes by sex and weight class. In addition to providing typical characteristics of such high caliber athletes, the study may aid in a more objective assessment of athlete weight class suitability. Further, Wang and Zhao utilized a novel approach to prescribing high-intensity interval training zones using anaerobic power reserve, which may create a more homogenous training stimulus and adaptation, but likely occurs independent of measurements of testosterone, cortisol or their ratio.

Collectively, the articles published in this research topic advance our knowledge in the field of rowing training and performance. Perspective and novel insight into the human, equipment, and on-water factors that may enhance or hinder rowing performance are highlighted. As always, these studies raise more questions that will hopefully spur on further research in the area.
